# Initial evaluation of prospective cardiac triggering using photoplethysmography signals recorded with a video camera compared to pulse oximetry and electrocardiography at 7T MRI

**DOI:** 10.1186/s12938-016-0245-3

**Published:** 2016-11-24

**Authors:** Nicolai Spicher, Markus Kukuk, Stefan Maderwald, Mark E. Ladd

**Affiliations:** 1 Department of Computer Science, University of Applied Sciences and Arts Dortmund, Emil-Figge-Str. 42, 44227 Dortmund, Germany; 2 Erwin L. Hahn Institute for Magnetic Resonance Imaging, University Duisburg-Essen, Kokereiallee 7, 45141 Essen, Germany; 3 Division of Medical Physics in Radiology, German Cancer Research Center, Im Neuenheimer Feld 280, 69120 Heidelberg, Germany

**Keywords:** MRI, Cardiac triggering, In-bore cameras, Pulse oximetry, Electrocardiography, Contact-free methods, Remote vital sign measurement

## Abstract

**Background:**

Accurate synchronization between magnetic resonance imaging data acquisition and a subject’s cardiac activity (“triggering”) is essential for reducing image artifacts but conventional, contact-based methods for this task are limited by several factors, including preparation time, patient inconvenience, and susceptibility to signal degradation. The purpose of this work is to evaluate the performance of a new contact-free triggering method developed with the aim to eventually replace conventional methods in non-cardiac imaging applications. In this study, the method’s performance is evaluated in the context of 7 Tesla non-enhanced angiography of the lower extremities.

**Methods:**

Our main contribution is a basic algorithm capable of estimating in real-time the phase of the cardiac cycle from reflection photoplethysmography signals obtained from skin color variations of the forehead recorded with a video camera. Instead of finding the algorithm’s parameters heuristically, they were optimized using videos of the forehead as well as electrocardiography and pulse oximetry signals that were recorded from eight healthy volunteers in and outside the scanner, with and without active radio frequency and gradient coils. Based on the video characteristics, synthetic signals were generated and the “best available” values of an objective function were determined using mathematical optimization. The performance of the proposed method with optimized algorithm parameters was evaluated by applying it to the recorded videos and comparing the computed triggers to those of contact-based methods. Additionally, the method was evaluated by using its triggers for acquiring images from a healthy volunteer and comparing the result to images obtained using pulse oximetry triggering.

**Results:**

During evaluation of the videos recorded inside the bore with active radio frequency and gradient coils, the pulse oximeter triggers were labeled in 62.5% as “potentially usable” for cardiac triggering, the electrocardiography triggers in 12.5%, and the proposed method’s triggers in 62.5%. Evaluation of the angiography images demonstrated that under appropriate conditions the method is feasible to produce an image quality comparable to pulse oximetry.

**Conclusion:**

We conclude that cardiac triggering using the proposed method is technically feasible. However, for improved reliability the signal-to-noise ratio of the videos will have to be addressed by either replacing the camera sensor, improving the illumination, or by use of additional signal filtering techniques.

## Background

Synchronization between data acquisition and a subject’s cardiac activity is an essential part of many magnetic resonance imaging (MRI) protocols and is referred to as cardiac “gating” or “triggering”. The blood flow from the heart to the periphery results in pulsatile vessel dilation that leads to artifacts if unsynchronized imaging is applied. Therefore, cardiac triggering is used to acquire image data only at a distinct phase of the cardiac cycle, assuming that at these points in time, the physiology of interest is spatially aligned.

Along with self-gating techniques [1], the performance of measurement hardware for cardiac triggering has been investigated extensively, namely Doppler ultrasound [2], pulse oximetry (PO), and electrocardiography (ECG) [3]. In clinical practice, PO and ECG are often favored due to their good performance regarding the trade-off between accuracy and set-up time. PO obtains a blood volume measurement called a photoplethysmogram (PPG) by exploiting the fact that the light absorption of blood is associated with its volume. In transmissive PO, a contact probe is applied to the fingertip or earlobe that measures the changing attenuation of light throughout the cardiac cycle. In reflectance PO, the light reflected from the skin is measured. In either case, the rise and fall of the PPG signal is associated with systole and diastole, making it suitable for cardiac triggering [4]. However, for cardiac imaging PO PPG is of limited value due to trigger jitter and delayed arrival of the blood in the finger [5, 6]. ECG measures the cumulative electrical activity of the heart from electrodes attached to the patient’s skin. The acquired waveform exhibits distinct signal features that are associated with cardiac activity, thereby allowing triggering at the desired phase of the cardiac cycle [3].

Both techniques are subject to several limitations: There is a principal risk of using contact-based measurement hardware inside the MRI scanner bore because it may lead to surface heating and burns on the patient’s skin if it is electrically conducting [7]. Additionally, the sensitivity to noise of ECG increases drastically because of magnetohydrodynamic interferences, especially during ultra-high-field MRI [8]. The application of a contact-based PO probe is limited to certain areas of the body and bears the risk of failing due to patient movement or low perfusion of his/her hands. In order to overcome these limitations, a phonocardiogram (PCG) based on the sound of the beating heart has been recently proposed. A microphone, which is not affected by magnetohydrodynamic interferences, is attached to the skin above the heart and has been shown to outperform ECG and PO during 7 Tesla cardiac imaging [6, 9].

Nevertheless, all contact-based hardware devices require clinical personnel for their proper application and direct interaction with the patient, which is time-consuming and may result in patient discomfort. In order to avoid physical contact, a MRI-compatible camera can be used, which offers other advantages as well: the camera is potentially not affected by interfering artifacts such as magnetohydrodynamic interactions, excitation pulses, or gradient noise, bears no risk of heating on the patient’s skin, and can be applied to all not-covered skin regions. In the past, camera-based solutions for clinical challenges during MRI have already been established, e.g. head motion correction [10].

We propose a video-based, contact- and marker-free method for real-time cardiac triggering, where the camera can be situated inside the bore of a MRI scanner and does not require alterations to its existing hard- or software if the camera provides basic MRI compatibility. Our main contribution is an algorithm that estimates the current phase of the cardiac cycle from skin color variations and triggers the scanner accordingly. The recorded color variations contain a subtle cardiac signal, resulting from dermis deformation caused by transmural arterial pressure changes [11] that has a waveform similar to a PO PPG and could therefore be used as a replacement. In the following, we use the term “video-based PPG signal” (vPPG) to describe the skin color variations.

Our main research interest lies in ultra-high-field non-enhanced angiography (MRA), which requires relatively long scanning times of approximately 60 min [12]. Unfortunately, patients often suffer from decreasing perfusion of the upper extremities with increasing scanning time, which often also results in degraded PO signal quality. As already mentioned, in a high-field environment, ECG is not a feasible triggering method because of magnetohydrodynamic artifacts. Our proposed triggering method was specifically developed with the aim to overcome these limitations in the context of MRA; however, other non-cardiac MRI procedures at high field strength requiring long scanning times would probably also benefit from the method.

### Related work

Remote measurement of physiological parameters has recently come to the focus of attention due to advances in computer and camera hardware. Specialized approaches have been proposed that allow remote estimation of the cardiac activity using thermal imagery [13, 14] or illumination at distinct wavelengths [15, 16]. Furthermore, it has been shown that ambient illumination and off-the-shelf cameras are sufficient as well [17, 18]. Recently, McDuff et al. presented results which indicate that using the cyan–green–orange color channels instead of red–green–blue enhances results [19]. Moreover, it has been shown that the whole morphology of the PO PPG waveform can be captured [20]. With respect to challenges in clinical practice, several studies have been conducted that included recording of digital color video signals and signal processing, carried out offline [21–23]. Visualization algorithms have also been presented: Wu et al. published an algorithm for magnification of manually selected frequency bands in videos that can be used to visualize the blood perfusion of the skin [24]. Kamshilin et al. presented a method for the computation of false-color videos that visualize the propagation of the cardiac component of vPPG signals [25].

In the context of MRI, it has recently been shown that vPPG signals recorded by in-bore cameras contain sufficient cardiac signal. This was accomplished offline by applying frequency filtering, as shown by Maclaren et al. [26, 27], or by applying the frequency magnification of Wu et al., as has been demonstrated in our preliminary work [28]. Furthermore, we presented results of vPPG-based real-time heart rate measurement [29] and triggering (without MRI image acquisition) for one subject with a predecessor of the algorithm presented in this work [30]. To our knowledge, MR image acquisition based on video triggers has been proposed as a future direction [27] but has not been pursued to date.

## Methods

### Overview

**Fig. 1:**
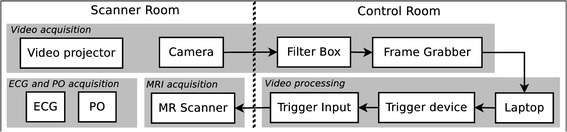
Schematic diagram of the proposed experimental set-up. The individual components (*text boxes*) are arranged in groups (*grey boxes*) indicating the subsection (*italic text*) of the manuscript. Components are connected using cable connections (*arrows*)

The proposed method for vPPG-based triggering consists of seven components following a linear workflow from the camera to the MRI scanner. Figure 1 shows a diagram of the experimental set-up: the video camera signal is sent from the scanner room to the control room into a filter box. Subsequently, the camera signal is digitized by a frame grabber connected to a laptop (see “Video acquisition”). The proposed algorithm estimates the current phase of the cardiac cycle and sends a corresponding trigger to the scanner via a custom-built device (see “Video processing”). The method can be used during all MRI procedures where a camera can be installed inside the bore. In this work, it was applied in a whole-body 7T scanner during MRA of the lower extremity vessels (see “MRI acquisition”) in healthy volunteers (see “Study population”).

In a first experiment, we recorded 120 min of video material as well as ECG and PO triggers (see “ECG and PO acquisition”) from eight subjects in and outside the scanner, with and without active radio frequency and gradient coils. Using synthetic vPPG signals derived from the videos and optimization, the best parameter configuration of the algorithm was determined (see “Algorithm parameter optimization”). Building upon these results, triggers from the proposed method were used for MRA image acquisition in a single healthy subject.

The performance of the proposed method was evaluated threefold (see “Results”): (1) The method was applied with optimized algorithm parameters on the videos recorded during the first experiment. As it was not possible to determine a one-to-one correspondence (bijective mapping) between vPPG, PO, and ECG triggers, they were compared qualitatively. The reason lies in the fact that for each triggering method, signal degradation due to the aforementioned reasons can occur at random points in time. (2) Using the vPPG and PO triggers from the second experiment, a one-to-one correspondence was ensured and a quantitative analysis of the triggers was performed by computing trigger delay and jitter. (3) The MRA images acquired by these triggers were visually compared.

### Video acquisition

Two requirements for an accurate estimation of the cardiac component in vPPG signals have been reported consistently: the cardiac signal is most intense in the green channel using RGB cameras [17, 22, 23] and most intense in the orange channel using RGBCO cameras [20]. These results indicate that a camera with an adequate spectral sensitivity is needed. Furthermore, intense and stable lightning is recommended to achieve satisfying results, especially in studies conducted in non-laboratory environments [21–23].

**Fig. 2:**
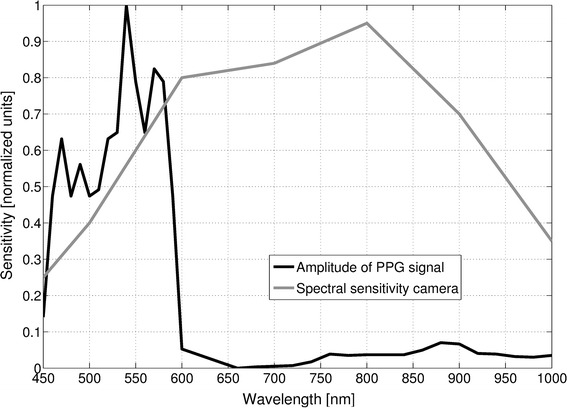
Wavelength-dependent sensitivity of MRI-compatible camera and amplitude of PPG signal. Relative amplitude of a PPG signal obtained in reflectance PO from one volunteer and spectral sensitivity of the camera used in this study. *Please note that the values of both curves were read by eyeballing diagrams found in the data-sheet of the camera and in literature* [36]

In this study, a commercially available MRI-compatible video camera (12M-i, MRC Systems, Heidelberg, Germany; 25 frames per second (FPS), 720 × 576 pixel) with a 1/3 inch monochrome sensor was used. Figure 2 shows the sensor’s spectral sensitivity and the relative amplitude of light reflected from human skin with respect to the wavelength during reflectance PO. As can clearly be seen, the PPG signal has its highest amplitude in the green-orange frequency band ($\approx450-600$ nm). In this band the camera exhibits a sensitivity up to 65%, which appears to be sufficient for obtaining an adequate vPPG signal as our previous works have shown [29, 30]. Maclaren et al. used a monochrome in-bore camera system, usually used for motion correction, with similar characteristics and obtained a vPPG signal with sufficient SNR as well [27].

**Fig. 3:**
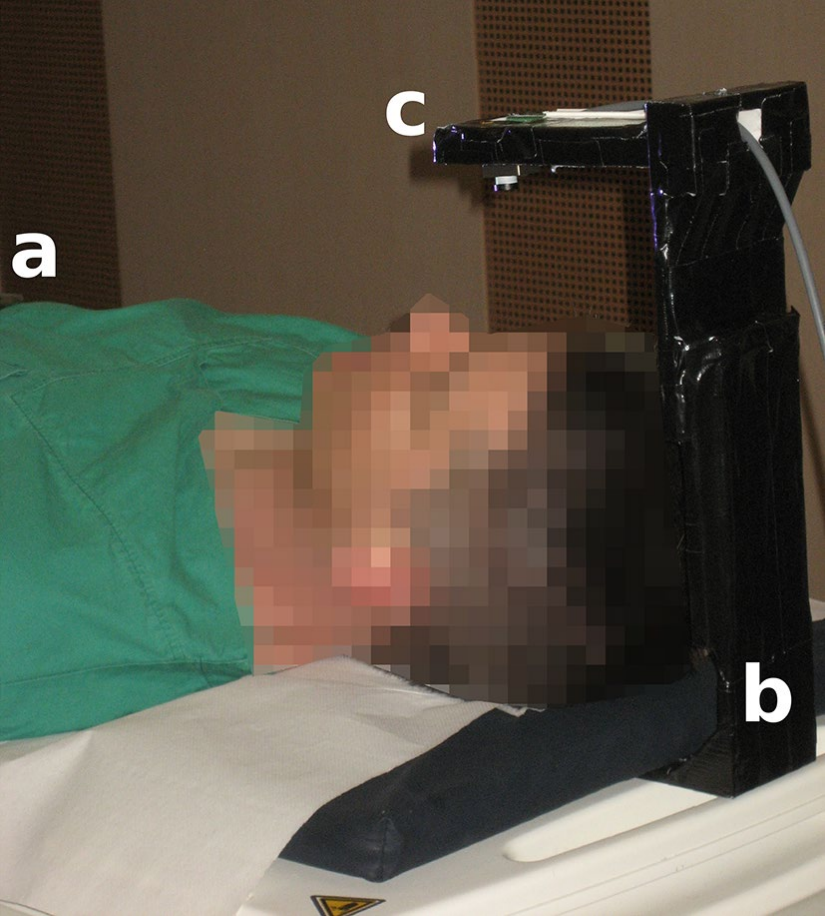
Hardware set-up of the proposed method. Set-up during experiments outside the bore showing a volunteer (**a**), the custom-built stand (**b**) and the MRI-compatible camera (**c**)

Figure 3 shows the camera set-up outside the bore. The camera was aimed at the subject’s forehead because it is easily accessible, well-perfused, and requires no additional preparations (e.g. hair removal). To satisfy the illumination requirements, we did not apply LED illumination directed towards the forehead as it increases patient discomfort, but instead an off-the-shelf video projector (LC-X71, EIKI Deutschland GmbH, Idstein, Germany; 5500 ANSI Lumens) was utilized, which is usually used for functional MRI stimulus delivery. White light was emitted by the projector from the rear towards the front of the bore. A filter box, containing low-pass filters that suppress high-frequency signal components introduced by the scanner, was provided by the camera vendor and set up accordingly. The frame grabber used was an off-the-shelf product (video processor unit: SC8113, Silan Microelectronics, Hangzhou, China).

### Video processing

**Fig. 4:**
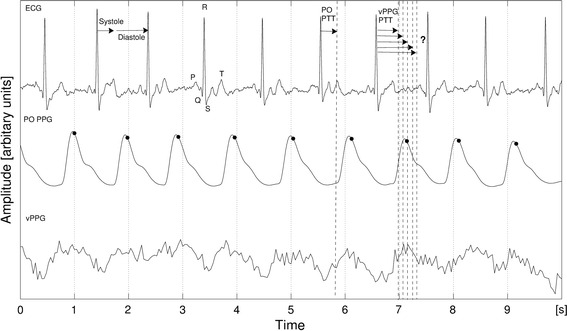
Relationship between ECG, PO PPG and vPPG signals. Typical fiducial points of the ECG and their position relative to the cardiac cycle are shown. The systole lasts from the R-wave to the T-wave, followed by the diastole until the next R-wave. The PO PTT (pulse transmit time) is usually measured from the ECG R-wave to the beginning of the next PO rise. Triggers computed by the MRI-vendor-provided software on the basis of the PO signal are marked by *black dots*

The proposed algorithm for cardiac triggering relies on the fact that color variations of a subject’s skin allow for remote estimation of his/her cardiac activity. Figure 4 shows a subject’s ECG signal (top) as well as a PPG signal obtained by transmissive finger PO (center) and a vPPG signal recorded with the MRI-compatible camera (bottom). The latter is obtained by spatial averaging of all pixels in a region-of-interest (ROI) which covers the subject’s skin. In the raw video, this signal is not visible to the naked eye due to its small amplitude but spatial averaging increases the signal-to-noise ratio (SNR) and reveals a subtle pseudo-periodic waveform similar to PO PPG that is associated with the subject’s cardiac activity.

For prospective cardiac triggering, the duration until the next maximum of the vPPG waveform has to be estimated from the current point in time. After this duration has passed, the trigger has to be issued to the MRI scanner. Similar to the short-time Fourier Transform (STFT), in our algorithm a time window is applied to the vPPG waveform at the current point in time (present) and previous signal values (past), covered by the window, are used to estimate the duration until the next maximum (future). This prediction only holds if the duration of the current cardiac cycle is similar to the durations of the preceding cardiac cycles covered by the time window. Therefore, the performance of the algorithm depends on the subject’s heart rate variability (HRV) and the occurrence of abnormal cardiac events, such as extrasystoles.

As can be seen in Fig. 4, when using finger PO PPG signals for cardiac triggering, the time of the signal’s maximum is used as a trigger because it approximately corresponds to the diastole, which is a result of the delay between the depolarization of the heart’s ventricles and the arrival of the arterial pulse wave at the location of PPG measurement called the pulse transit time (PPG PTT) [4]. No exact information regarding the PTT for vPPG signals measured from the forehead using our set-up is available. McDuff et al. observed the relationship between vPPG signals measured from the face and finger PO and showed high agreement in peak and interval timings [19], but they used different hardware and algorithms. Furthermore, the proposed algorithm runs in real time, which may eventually require adjustments of the computed durations by subtracting the computation time. To account for these aspects, an offset parameter is added that allows manually modifying the computed duration until the next maximum.

#### Algorithm

The basic idea of the proposed algorithm is to use the time shifting property of the Fourier Transform to estimate the duration until the next maximum of the vPPG signal’s cardiac component. The algorithm uses the current vPPG signal values as input, estimates the frequency of the cardiac component from the spectrum, and uses its phase to estimate the duration until it will reach the next maximum. The algorithm waits for the computed duration, eventually adjusted by the already mentioned parameter, and then issues a trigger to the MRI scanner.

The proposed method relies on a fundamental assumption: The subject’s heart is in sinus rhythm and has a rate in the range of $hr_{min}=48$ to $hr_{max}=180$ beats-per-minute (bpm), corresponding to 0.8 and 3 Hz. Input parameters of the algorithm are a rectangular ROI that covers most of the subject’s forehead and three integers $M > 0$, $N > 0$, and *T*, where the latter may assume positive or negative values: *N* is the number of processed frames, *M* is used for averaging, and *T* if an offset added to the computed duration until the next trigger. Due to the summation of the values in the ROI, the algorithm is intrinsically robust to smaller subject motion.

In the first step of the algorithm, digital frames $I:\mathbb {N}^2 \rightarrow \mathbb {N}$ are obtained successively from the frame grabber, are cropped to the predefined ROI with integer coordinates (*m*, *n*), and are stored in a list:

1 $$\begin{aligned} I_0(m,n),~I_1(m,n),~I_2(m,n),~I_3(m,n),~ \ldots \end{aligned}$$

Using parameter *N*, an interval is defined that is used to fetch all consecutive frames from $I_a$ to $I_b$:

2 $$\begin{aligned}{}[a, b] = \{x \in \mathbb {N}_0 ~|~ (a \le x \le b) ~ \wedge ~ (b-a={N}-1) \} \end{aligned}$$

The interval is chosen such that the newest frames are selected. To account for zero padding, another interval is defined

3 $$\begin{aligned}{}[c,d] = \{x \in \mathbb {N}_0 ~|~ (c \le a \le x \le b \le d) ~ \wedge ~ (d-c=2^p-1) \} \end{aligned}$$

where *p* is the smallest integer such that $2^p > {N}$. The following steps of the algorithm are executed based on both intervals. Subsequently, the algorithm repeats itself by defining new intervals and performing the following steps again.

The vPPG signal $f_{s}$ is computed from zero-padded color intensity variations of the forehead

4 $$\begin{aligned} f_{s}: \mathbb {N}_0 \rightarrow \mathbb {N}_0, t \mapsto {\left\{ \begin{array}{ll} \sum \limits _{m} \sum \limits _{n} I_{t}(m,n) &{} \quad \text {if } t \in [a,b] \\ 0 &{} \quad \text {otherwise} \end{array}\right. }~, t\in [c,d]. \end{aligned}$$

Additionally, the output of $f_{s}$ is normalized to lie between 0 and 1. To account for spectral leakage, a Hamming window

5 $$\begin{aligned} f_h: \mathbb {N}_0 \rightarrow \mathbb {R}, t \mapsto 0.54 - 0.46 \cos ( {2 \pi t }/{(2^p-1)} ), t \in [0,2^p-1] \end{aligned}$$

is applied when computing the Fourier spectrum of the vPPG signal

6 $$\begin{aligned} F: \mathbb {N}_0 \rightarrow \mathbb {C}, k \mapsto \sum \limits _{t} f_{s}(t) f_h (t) e^{-j 2 \pi t k / 2^p}, k\in [0,2^p-1]. \end{aligned}$$

As $f_{s}(t) f_h (t)$ is real and therefore *F*(*k*) is conjugate symmetric, only the first half interval $[0,2^{p-1}-1]$ of the spectrum is considered. The function

7 $$\begin{aligned} f_{hz}: \mathbb {N}_0 \rightarrow \mathbb {R}[\text {Hz}], k \mapsto {k k_s}/{2^p}, k \in [0,2^{p-1}-1] \end{aligned}$$

is used where $k_s$ represents the sampling rate of the camera, to compute the frequency associated to the bins of the spectrum. According to the underlying assumption of the algorithm regarding the subject heart rates, only frequencies [*u*, *v*] are considered such that $u,v \in [0,2^{p-1}-1]$, and $f_{hz}(u) \approx 0.8 \text{ Hz }$ as well as $f_{hz}(v) \approx 3\text{ Hz }$. The maximum peak in the spectrum is detected by

8 $$\begin{aligned} k_{peak} := \arg \max _{y \in [u,v]} | F(y) | \text {~where~}| F(y) | = \sqrt{\mathfrak {R}(F(y))^2 + \mathfrak {I}(F(y))^2} \end{aligned}$$

where $\mathfrak {R}$ and $\mathfrak {I}$ represent the real and imaginary part of the Fourier spectrum, respectively. Additionally, $k_{peak}$ is averaged over the last *M* computations to account for outliers. The frequency associated to the maximum peak, i.e. the heart rate of the subject, is obtained by

9 $$\begin{aligned} \gamma _{peak} \text{[Hz] } := f_{hz} \big ( k_{peak} \big ) \end{aligned}$$

and the corresponding phase by

10 $$\begin{aligned} \varphi _{peak} \text{[rad] } := \arctan \bigg ( \frac{\mathfrak {I}\big ( F (k_{peak}) \big )}{\mathfrak {R}\big ( F ( k_{peak} ) \big )} \bigg ). \end{aligned}$$

Finally, the time until the next trigger is calculated:

11 $$\begin{aligned} \Delta [s] = T \pm {\left\{ \begin{array}{ll} \mid \varphi _{peak} / (2 \pi \gamma _{peak}) \mid &{} \quad \text{ if } \varphi _{peak}<0 \\ {\gamma _{peak}}^{-1} - \mid \varphi _{peak} / (2 \pi \gamma _{peak}) \mid &{} \quad \text{ otherwise } \end{array}\right. } \end{aligned}$$

where the parameter *T* is used to manually increase or decrease this value. After computation of $\Delta$, the algorithm checks if the time elapsed since the previous trigger was larger than the minimum delay (${hr_{max}[bpm]} = {60\,\text{s}}/{180}=0.333$ s) between two heart beats according to the algorithm’s assumption. If that is the case, the algorithm waits for $\Delta$ seconds and sends a trigger subsequently. If the maximum delay (${hr_{min}[bpm]} = {60\,\text{s}}/{48}=1.250$ s) is exceeded without sending of a trigger, one is enforced. As stated before, the algorithm then returns to the step of interval [*a*, *b*] selection.

The proposed algorithm was implemented in C$^{++}$11 (gcc v4.8.4) using standard library features such as the multi-threading capabilities of the std::thread class. The Open Source Computer Vision library (v2.4.9) [31] as well as the ROOT library (v5.34) [32] were used.

#### Trigger device

A custom-built, low-cost device for triggering of the MRI scanner was developed based on an open source development board (Arduino Nano, Arduino, Turin, Italy; AT-mega328 microcontroller) which is connected to a laptop via USB connection. Hardware by this vendor has already been used successfully in a multi-purpose open-source trigger platform [33]. If a trigger signal is invoked by the algorithm, it is forwarded from the device to the MRI scanner trigger input.

### MRI acquisition

Experiments were performed on an ultra-high-field whole-body MRI system (MAGNETOM 7T; Siemens GmbH, Erlangen, Germany) using the MRA setup as described by Fischer et al. [12] with a 16-channel transmit/receive body coil [34].

### ECG and PO acquisition

All measurement hardware was provided by the MRI vendor. Measurements were sent via Bluetooth wireless technology to the vendor-provided software. PO data was discretized with a sampling rate of 50Hz and ECG data was discretized with a sampling rate of 400 Hz.

In the first experiment, the PO probe was attached to the left index finger, and three ECG electrodes were applied on the sternum and the left side of the thorax. In the second experiment, only the PO probe was used.

### Study population

First experiment: Eight healthy volunteers [gender: 2 female, age: 32.0 ± 4.5 years, weight: 74.5 ± 11.5 kg, height: 179.1 ± 8.5 cm; mean ± standard deviation (sd)] with different ethnic origins (6 Europe, 1 West Asia, 1 South Asia) and therefore different skin characteristics were examined.

Second experiment: One healthy volunteer (gender: male, age: 34 years, weight: 80 kg, height: 172 cm) with European origin that did not take part in the first experiment was examined.

Each subject was informed about the examination procedure by a physician and written informed consent was obtained before the examination.

### Measurement protocol

The first experiment was conducted to obtain video and trigger data for parameter optimization. Therefore, MRI image acquisition was performed temporarily during the experiment to analyze the influence of noise sources such as excitation pulses or gradient vibrations on the different triggering methods, but images were not stored. In the second experiment, actual acquisition of MRA images was performed.

The first experiment was conducted as follows: (1) patient table in home position at approximately 0.3 T with room illumination; (2) patient table in isocenter of scanner bore at 7 T with illumination from an off-the-shelf video projector without activity of the gradient and radio frequency coils; (3) patient table in isocenter of scanner bore at 7 T with illumination from an off-the-shelf video projector with activity of the gradient and radio frequency coils. The latter was performed according to our MRA protocol [12], and PO was used as the triggering method. The stages were performed in order (1) (2) (3) with duration of 5 min each. Between each individual stage, video and data acquisition were halted, resulting in three recordings for each of the eight subjects and 24 recordings in total. Figure 3 shows the set-up during the first stage.

In the second experiment, a volunteer, who was not part of the cohort from the first experiment, underwent MRA of the lower extremity vessels. The transmit/receive coil was positioned on the transition from thigh to the kneecap so that the obtained images contain differing anatomical structures. Using the MRI vendor-provided software, maximum intensity projection (MIP) images were reconstructed in coronal orientation using the following order of triggering methods: PO PPG, vPPG, PO PPG, vPPG. PO PPG was used as ground truth and the alternating order was chosen to account for HRV of the subject.

### Algorithm parameter optimization

A suitable configuration of the algorithm’s parameters could not be deduced from physiological or technical considerations; for example, increasing parameter *N* results mathematically in a Fourier spectrum with higher resolution, but now the transformed vPPG signal also spans more cardiac cycles, which results in averaging of the computed phase. Additionally, a reasonable value for parameter *T*, which can be used to modify the computed durations until issuance of a trigger, is unknown. Therefore, optimization over the algorithm parameters was performed. Due to the rather low number of subjects and recorded videos in this study, we did not perform an exhaustive optimization over *M*, *N*, and *T* using the video data, but instead we applied a two-step method by optimizing first over parameters *M* and *N* using a large set of synthetic signals and video recorded in the first experiment, followed by optimization over parameter *T* using the videos only:

In the first optimization over *M* and *N*, two sets of synthetic signals were constructed. The first set was based on realistic HRV values, derived from an expert-annotated ECG database and realistic SNR values, derived from the video data of the first experiment. The second set represented the control group with the same SNR characteristics but without HRV. An objective function was minimized over parameters *M* and *N* and was formulated as the sum of errors resulting from the difference between computed and ground-truth trigger times. We used the results obtained with the second set to verify our assumptions about parameters *M* and *N*. Results that were obtained with the first set were used as a guideline for choosing both parameters. For this task, we introduced a new criterion based on visual histogram comparison and chose the best performing parameter combination from the set of combinations provided by the guideline.

In the second optimization over *T*, synthetic signals could not be used since they do not contain physiological information regarding the PTT. Therefore, vPPG signals from the videos recorded during the first experiment and the best performing *M* and *N* configuration from the first optimization step were used. As the ECG triggers were in most cases unusable due to high-field artifacts, the PO triggers were chosen as ground truth. An objective function was minimized over parameter *T* and formulated as the error between cardiac cycle durations, i.e. the durations from trigger to trigger, computed by our method and from PO.

The first step of optimization was performed in MATLAB R2012a (MathWorks, Natick, MA, USA). The second step of optimization was performed using the mentioned real-time feasible algorithm.

#### Noise estimation from videos

Each video was processed individually and the vPPG signal $f_s$ was computed according to the algorithm described, but the interval [*a*, *b*] was chosen such that all frames of the videos were used. Zero padding was not applied and therefore $[a,b] = [c,d]$. Three types of SNR were estimated from each vPPG signal:

- $\mathrm {SNR_{VLF}}$: very low frequency noise [0,0.1] Hz
- $\mathrm {SNR_{LF}}$: low frequency noise [0.1,0.8] Hz
- $\mathrm {SNR_{HF}}$: high frequency noise [3,12.5] Hz

The cardiac information in the vPPG signal was assumed to lie between 48 and 180 bpm ([0.8, 3] Hz), which could contain healthy as well as pathological heart rates. Spectral components with a higher frequency are primarily a result of high-frequency camera sensor noise. Spectral components with a lower frequency than the cardiac signal are divided into LF and VLF components. The first could contain physiological components, such as respiration, while the latter do not.

The SNR between the cardiac signal $f(t)_ {HR}$ and each type of noise ($f(t)_{VLF}$, $f(t)_{LF}$, $f(t)_{HF}$) was computed by using at first cut-off filters in frequency domain with passbands according to the corresponding intervals and then the SNR of the filtered signal was computed by

12 $$\begin{aligned} {\mathrm {SNR_{LF}\,[dB]} = 10 \log _{10} \left( \frac{{\Sigma _{n=0}^{N-1}f(t)_{HR}^2}{/N-1}}{{\Sigma _{n=0}^{N-1}f(t)_{LF}^2}{/N-1}} \right) } \end{aligned}$$

given here for LF noise. Additionally, the distribution of the noise data stored in $f(t)_{VLF}$, $f(t)_{LF}$, and $f(t)_{HF}$ was analyzed. Visual inspection showed that in most cases $f(t)_{LF}$ and $f(t)_{HF}$ accurately resemble a logistic distribution

13 $$\begin{aligned} f_{log}(t) = \frac{e^{-\frac{t-\mu _{log}}{\sigma _{log}}}}{\sigma _{log}\left( 1+e^{-\frac{t-\mu _{log}}{\sigma _{log}}}\right) ^2} \end{aligned}$$

The noise in $f(t)_{VLF}$ did not follow a recognizable distribution and therefore it was assumed to be normally distributed

14 $$\begin{aligned} f_{norm}(t) = \frac{1}{\sqrt{2\pi \sigma _{norm}^2} } e^{ -\frac{(t-\mu _{norm})^2}{2\sigma _{norm}^2} } \end{aligned}$$

Additionally, the Kolmogorov-Smirnov test (KS, $\alpha = 0.01$, *H*0: Input comes from distribution) was used to further confirm that the noise follows the chosen distributions and they were fitted to $f(t)_{VLF}$, $f(t)_{LF}$, and $f(t)_{HF}$ using the maximum likelihood operator.

#### Synthetic signal generation

It was assumed that skin color variations from the outflow of blood (low color intensity) to the influx of blood (high color intensity), and the reoccurring outflow of blood (low color intensity) could be simulated by using the cosine function. Cardiac cycle durations were acquired from the annotated “Normal Sinus Rhythm RR Interval Database” (http://www.physionet.org/physiobank/database/nsr2db/ [35]), which contains data from 54 healthy subjects. As fiducial points, the duration between successive R waves, i.e. the RR intervals, were used.

Two sets of synthetic signals were constructed with the same sampling rate as the videos (25 Hz): For all subjects in the ECG database, signal segments representing exactly one period of a cardiac cycle using $f_{cc} : \mathbb {R} \mapsto \mathbb {R}, t \rightarrow -\cos (2\pi ~1/t^{RR}_n~t), t \in [0, t^{RR}_n]$ where $t^{RR}_n$ was the *n*-th RR interval from the recording, were appended until a signal with a duration of five minutes was obtained. This procedure was repeated for the second synthetic signal set signals without HRV and therefore a constant heart rate of 60 bpm ($t^{RR}_n=1$), resulting in two sets with each containing 4.5 h of synthetic vPPG signals.

Subsequently, VLF, LF, and HF noise were added. For each synthetic signal, the noise characteristics were chosen pseudo-randomly from the fitted values of one of the 24 videos.

#### Parameter optimization

In the first optimization step, a modified version of the algorithm for cardiac triggering was executed on both sets of synthetic vPPG signals with the parameters *N* $\in$ $\{ 50,$ 100,  150,  200,  250,  300,  350,  400,  450,  500,  550,  $600\}$, *M* $\in$ $\{ 1,$ 5,  10,  25,  50,  100,  150,  200,  250,  $300\}$ and fixed ${T}=0$. A grid search approach was used that executed the algorithm on each signal with each parameter pair from the Cartesian product of *M* and *N*. Subsequently, the parameter combinations associated with the lowest error when processing the synthetic vPPG signals with HRV were applied to the vPPG signals recorded during experiments. The best performing combination was chosen by visually comparing the histograms of trigger-to-trigger durations computed by the algorithm to PO trigger-to-trigger durations.

The modifications of the algorithm for parameter optimization were as follows: Instead of obtaining accumulated pixel intensities of video frames, the function $f_s$ returned the values of the synthetic signals. Additionally, the algorithm did not wait to trigger the MRI scanner but stored the $\Delta$ value without delay and zero-padding was not applied ($[a,b]=[c,d]$) since it would bias the comparability of different *N* values.

After obtaining an optimal choice of *M* and *N*, the recorded videos and trigger data were used to optimize over parameter *T* in the second optimization step. The proposed algorithm was executed without modifications on all videos with fixed *M* and *N* and a variable ${T} \in \{ -300,\,-200,\,-100,\,0,\,100,\,200,\,300\}$.

## Results

### Noise estimation from videos

**Fig. 5:**
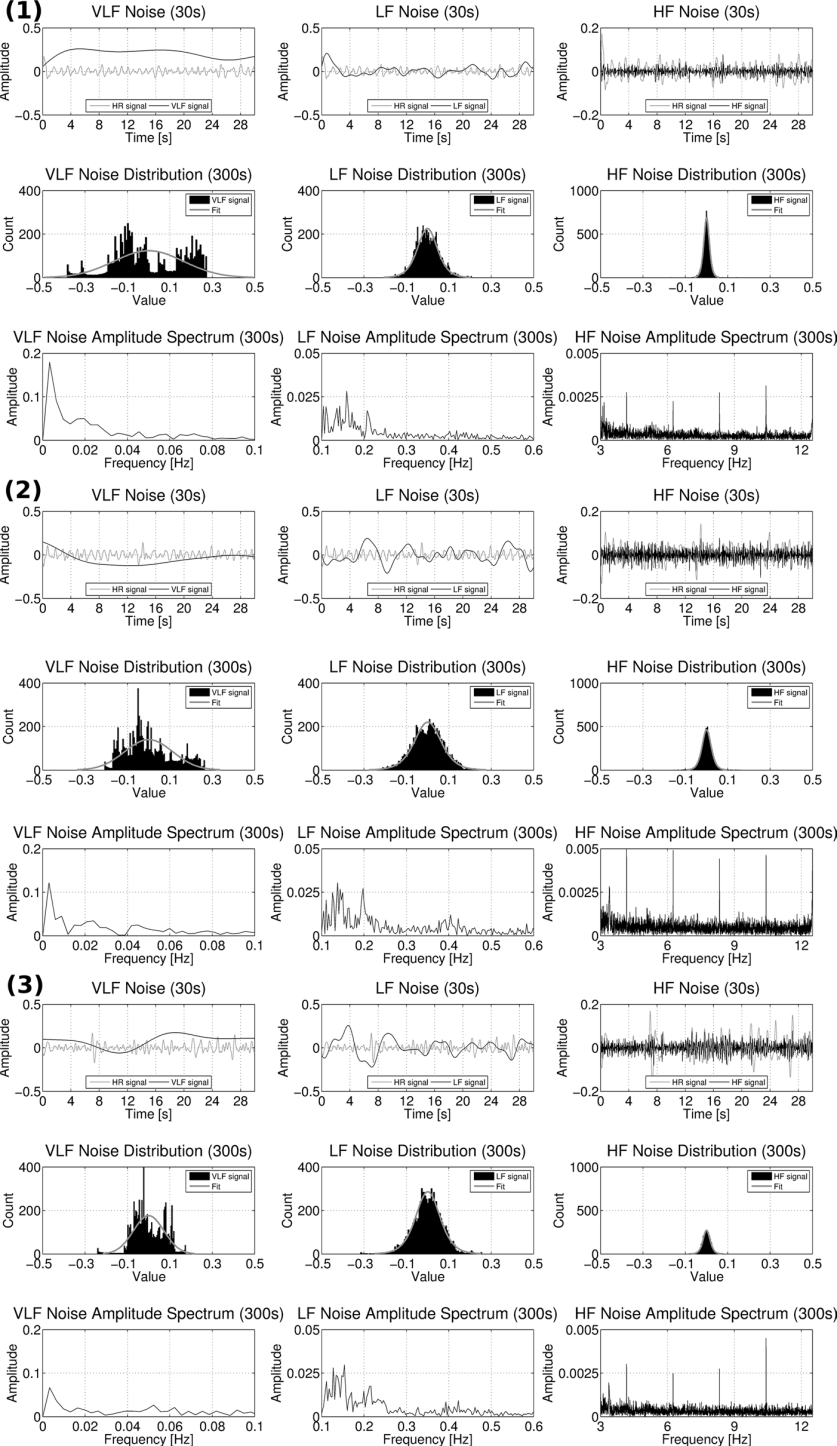
Characteristics of VLF, LF and HF noise. Shown here are all results from one subject obtained during stages (**1**) outside the MRI bore, (**2**) inside the MRI bore without imaging, and (**3**) inside the MRI bore with imaging. Inside each section, the first row shows 30 s of VLF, LF, and HF noise extracted from the vPPG signal. As reference, the cardiac component of the vPPG signal is shown in* gray color*. The second and third row show the histograms and one-sided Fourier spectra from the entire signal with a duration of 5 min

Figure 5 shows results of vPPG noise estimation from the videos of a subject recorded in stages (1), (2), and (3), respectively. As can be seen clearly from the signals in time domain (first rows), the VLF noise has the lowest SNR compared to the cardiac signal, followed by the LF and HF noise, respectively. The histograms (second rows) show that while the HF and LF noise is quite consistent with the logistic distribution, the VLF is not described as adequately by the normal distribution during all stages. The Fourier spectra (third rows) show that the LF signal contains a signal portion around 0.2 Hz that could be a result from respiration of the subject. Additionally, the HF signal contains high-frequency harmonics in very narrow frequency bands that are most presumably a result of the used camera hardware.

Table 1 shows results for the videos of all subjects: In 23/24 videos, the SNR is lowest for the VLF noise, followed by the LF and the HF noise, respectively. Furthermore, the KS Test rejects the null hypothesis always for the VLF noise, 21 times for the LF noise, and 12 times for the HF noise. In 6/8 subjects, one or both of the in-bore recordings in stage (2) and (3) had a lower SNR for all three types of noise compared to the recordings outside the bore in stage (1). In 6/8 subjects there is no clear signal with a superior or inferior SNR when comparing results from stage (2) and (3).

**Table 1:** Noise properties from videos recorded during the measurement protocol

Result	Noise	1/(1)	1/(2)	1/(3)	2/(1)	2/(2)	2/(3)	3/(1)	3/(2)	3/(3)
vPPG signal computed from video of subject/stage										
SNR (dB)	VLF	−9.3838	−11.1829	−7.4733	−9.1363	−15.2723	−15.5896	−15.8005	−8.2305	−7.8545
	LF	−4.1574	−9.5661	−9.0184	−0.6905	−1.1406	−0.0327	−6.3080	−4.4844	−7.4882
	HF	8.8457	6.0230	5.6720	7.0804	3.8714	5.1022	4.4189	4.4099	3.4681
Estimated $\mu$	VLF	0.0000	0.0000	0.0000	0.0000	0.0000	0.0000	0.0000	0.0000	0.0000
	LF	0.0004	−0.0005	0.0039	0.0012	0.0004	0.0002	−0.0011	0.0007	0.0010
	HF	0.0001	0.0001	−0.0001	0.0000	0.0001	0.0000	0.0002	0.0002	0.0001
Estimated $\sigma$	VLF	0.1288	0.1201	0.0994	0.1251	0.2056	0.1344	0.1644	0.1126	0.0719
	LF	0.0399	0.0598	0.0709	0.0259	0.0214	0.0128	0.0313	0.0418	0.0382
	HF	0.0081	0.0092	0.0120	0.0105	0.0121	0.0068	0.0087	0.0142	0.0111
KS test	VLF	1	1	1	1	1	1	1	1	1
	LF	1	1	1	1	1	1	1	1	0
	HF	1	0	0	0	0	0	0	0	1

		4/(1)	4/(2)	4/(3)	5/(1)	5/(2)	5/(3)	6/(1)	6/(2)	6/(3)
SNR (dB)	VLF	−16.6716	−21.0377	−7.7598	−19.9921	−15.5100	−13.4868	−5.6413	−7.2446	−19.7351
	LF	−2.7974	−4.1851	−3.1127	−7.6886	−5.3285	−6.5990	0.7468	0.9326	−5.9409
	HF	7.7905	5.0893	4.8462	6.2689	7.5451	7.3158	9.0495	8.1937	6.0176
Estimated $\mu$	VLF	0.0000	0.0000	0.0000	0.0000	0.0000	0.0000	0.0000	0.0000	0.0000
	LF	−0.0003	−0.0002	0.0004	−0.0003	−0.0002	0.0011	0.0008	−0.0000	−0.0001
	HF	0.0001	0.0004	0.0009	0.0001	−0.0000	0.0000	0.0000	−0.0001	−0.0000
Estimated $\sigma$	VLF	0.2149	0.2689	0.1253	0.2048	0.1579	0.1702	0.1257	0.1356	0.2005
	LF	0.0203	0.0198	0.0419	0.0260	0.0277	0.0449	0.0343	0.0307	0.0163
	HF	0.0063	0.0072	0.0165	0.0044	0.0058	0.0083	0.0123	0.0127	0.0049
KS test	VLF	1	1	1	1	1	1	1	1	1
	LF	1	1	1	1	0	1	1	1	1
	HF	1	1	1	1	1	0	1	0	0

		7/(1)	7/(2)	7/(3)	8/(1)	8/(2)	8/(3)			
SNR (dB)	VLF	−5.7181	−11.7821	−6.2986	−4.8782	−9.8592	−8.6507			
	LF	0.4626	−0.9377	2.5547	−1.0125	−1.0125	−4.1720			
	HF	9.0742	7.0814	7.1712	7.3655	6.2019	6.2604			
Estimated $\mu$	VLF	0.0000	0.0000	0.0000	0.0000	0.0000	0.0000			
	LF	0.0008	0.0001	−0.0009	0.0019	0.0019	0.0014			
	HF	0.0001	0.0003	0.0005	0.0002	0.0001	0.0002			
Estimated $\sigma$	VLF	0.1200	0.1910	0.1481	0.1077	0.1284	0.1047			
	LF	0.0329	0.0299	0.0306	0.0398	0.0491	0.0369			
	HF	0.0116	0.0123	0.0179	0.0141	0.0109	0.0102			
KS test	VLF	1	1	1	1	1	1			
	LF	0	1	1	1	1	1			
	HF	1	1	1	1	0	0			

All values are rounded to the fourth decimal place. $\mu$ and $\sigma$ are obtained by either fitting the logistic distribution (Eq. 13) to histograms of the $f(t)_{LF}$ and $f(t)_{HF}$ noise or fitting the normal distribution (Eq. 14) to histograms of $f(t)_{VLF}$ . The result of the KS test is 1 if the test rejects the null hypothesis at $\alpha =1\%$ and 0 otherwise

**Fig. 6:**
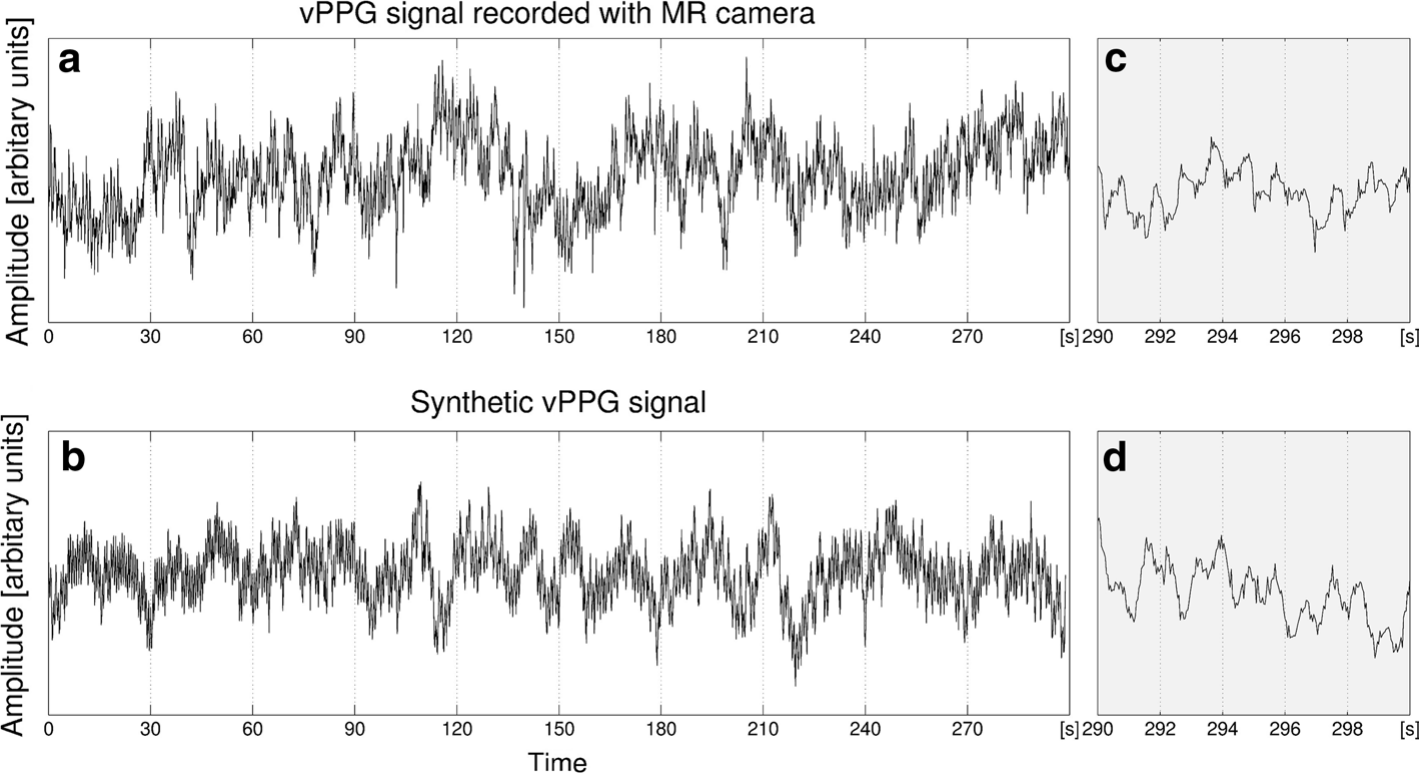
Real vPPG signal recorded using the MRI-compatible camera and synthetic vPPG signal with the same noise characteristics. **a** Shows a complete vPPG signal obtained from a video recorded during the first stage of the measurement protocol. **b** Shows a synthetic vPPG signal computed from RR intervals from the ECG database with VLF, LF, and HF noise estimated from the signal shown in **a**. The last 10 s of signals **a** and** b** are shown in **c** and **d**, respectively

Figure 6 shows a vPPG signal and a synthetic signal computed from the same noise characteristics and it can be seen that they are comparable. All synthetic signals were inspected by eyeballing and it was concluded that they capture the noise characteristics sufficiently.

### Parameter *M* and *N* optimization

**Fig. 7:**
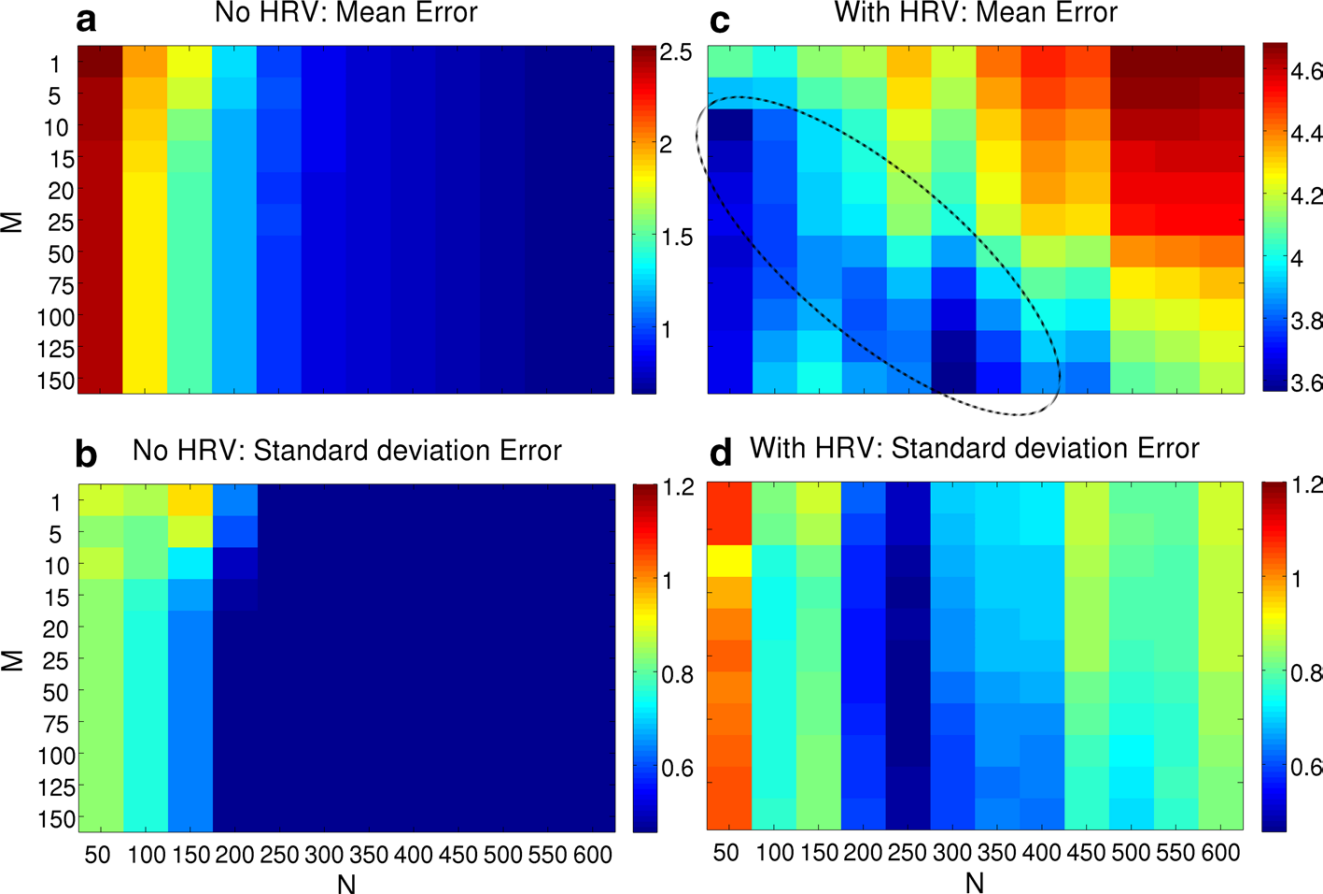
Algorithms performance on both types of synthetic vPPG signals. Results of the synthetic signals without (*left*) and with HRV (*right*) are shown in column, respectively. The* first row* **a**, **c** shows the mean error, while the *second row* **b**, **d** shows sd. For each synthetic signal, computed trigger times were compared to ground truth by absolute differences and the averaged error was stored. After processing all signals, the mean (**a**, **c**) and sd (**b**, **d**) of the individual errors was stored in the matrix. A minimum valley is highlighted in** c**. Values are displayed in unit samples

For each synthetic vPPG signal and parameter combination, the absolute differences between the computed trigger points in time and the ground truth cosine maxima were averaged by arithmetic mean and stored as the corresponding error. Figure 7 displays the mean and sd values averaged over all synthetic signal mean errors separately for synthetic signals with (c+d) and without HRV (a+b). The signals without HRV were used as a control; increasing *M* and *N* should always result in a lower mean error. As can be seen, this is the case and *N* has a larger impact than *M*. When choosing the number of frames *N* larger then 250, the mean error is below one sample. For synthetic signals with HRV, this relationship does not hold. The mean error is inferior, with the best combination achieving a mean error of approximately 3.6 samples. The data exhibit a minimum error valley that reaches two minima: ($N = 50$, $M = 10$) and ($N = 300$, $M = 150$).

The sd was further analyzed, which showed that the results without HRV (b) are more robust and result in a value smaller than 0.5 for *N* larger than 200. For synthetic signals with HRV (d), the results on the right side ($N = 300$, $M = 150$, sd $= 0.58$) are more stable than the results on the left side ($N = 50$, $M = 10$, sd $= 0.91$) of the valley. In theory, using a lower *N* leads to more accurate results, but this approach is also prone to noise. Using a higher *N* averages the values over more cardiac cycles but is also more robust against outliers.

**Fig. 8:**
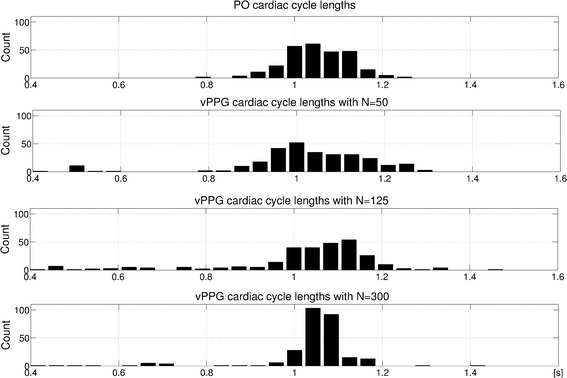
Histograms of cardiac cycle durations between subsequent triggers. The corresponding video has been obtained during stage (1). As can be seen, the algorithm produces a histogram which is too wide for N = 50, i.e. there are many too long/short cardiac cycle durations, and a histogram which is too narrow for N = 300, i.e. the cardiac cycle durations are averaged over several cardiac cycles. N = 125 results in a histogram more similar to PO

Based on these results, we introduced a criterion in order to find the best choice of parameters *M* and *N* drawn from the minimum error valley for non-synthetic vPPG signals. Both parameter configurations associated with the minima were chosen and the durations between the vPPG triggers and the PO-based triggers were visually compared. A representative result can be seen in Fig. 8. Neither parameter configuration that achieved best results for the synthetic signals [$N = 50$ (associated with 2 s of video data) and $N = 300$ (12s.)] is optimal for the video signals at hand. After visually analyzing the histograms of all videos, a value $N=125$ (5s.) was chosen for further use because in most cases it is more similar to the PO-based distribution. The influence of parameter *M* was rather small and $M=50$ was chosen because it approximately corresponds to the value in Fig. 7c at $N=125$.

### Parameter T optimization

**Fig. 9:**
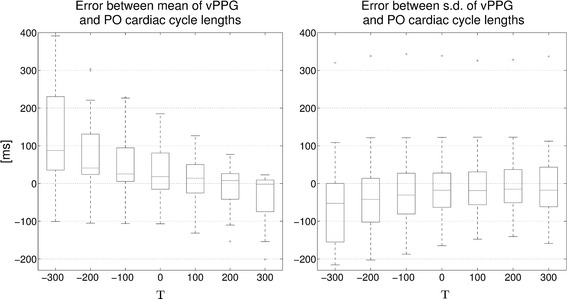
Algorithms performance with variable *T*. For each video, triggers were computed with the algorithm for $N = 125$, $M = 50$, and a variable *T* as indicated on the x-axis. For each video, the mean and sd of the durations between consecutive trigger points are computed for the vPPG and PO triggers. In the boxplots, differences between the mean and sd values of both methods are shown

After obtaining the optimal parameters $M=50$ and $N=125$, the algorithm was executed with this configuration on all videos in order to optimize over parameter *T*. For each video, the durations between consecutive vPPG triggers and between consecutive PO triggers were stored and the mean and sd values were compared. Figure 9 shows a boxplot computed from the error between both methods depending on *T*. As can be seen, the algorithm achieves poor results for negative *T* values. Apparently, shortening the duration until the next maximum leads to false, premature triggers in addition to the actual correct triggers. The best configuration is achieved when using $T=200$ ms. The $T=300$ ms configuration has a median closer to zero for the mean error, but it is also skewed towards more negative values and has an inferior sd error.

### Evaluation of vPPG-based triggering

#### Comparison of ECG, PO, and vPPG triggers

**Fig. 10:**
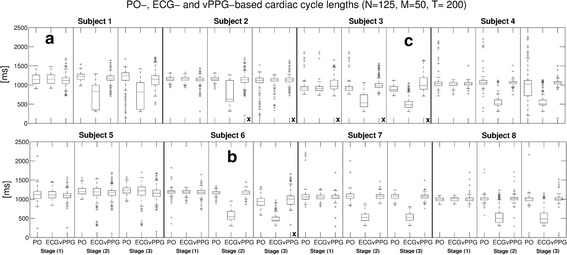
Algorithm performance with videos obtained in MRI scanner room. For each of the 24 videos, triggers were computed with the algorithm and parameters $N = 125$, $M = 50$, and $T = 200$. For each subject, the results from* stage* (*1*) (outside MRI bore),* stage* (*2*) (inside MRI bore without imaging), and* stage* (*3*) (inside MRI bore with imaging) are shown. Boxplots of intervals between consecutive triggers based on PO, ECG, and vPPG are shown. **a**, **b** and **c** mark videos that are shown in detail in Fig. 11. An x marks videos where the vPPG triggering has been labeled as unusable

After finding an optimal choice of all parameters ($M=50$, $N=125$, $T=200$), the algorithm was executed on the videos recorded during the first experiment. Figure 10 shows boxplots of the cardiac cycle durations, i.e. the durations between successive triggers, from all three trigger methods.

The cardiac cycle durations based on ECG and PO triggers were very similar outside the MRI scanner bore [stage (1)], while the vPPG cardiac cycle durations had a similar median but more outliers. Inside the bore, the PO remained stable without imaging [stage (2)], but it was distorted during imaging [stage (3)] in 3/8 subjects, presumably due to susceptibility to gradient vibrations of the MRI vendor-provided Bluetooth module. The accuracy of the ECG was degraded drastically during in-bore stages in 7/8 subjects due to magnetohydrodynamic interferences [8]. Regarding the vPPG triggers, the difference between experiments inside and outside the bore were not as severe because the camera was apparently not affected by the magnetic environment or gradient vibrations. Also, examination of the video frames did not reveal artifacts visible to the naked eye. Nevertheless, the number of outliers was increased for most in-bore videos, presumably due to inferior illumination conditions.

As a quantitative comparison of the different triggering methods was not possible because there is no one-to-one association between the triggers by the different methods, their performance was visually analyzed and classified qualitatively for each video into “potentially usable” for cardiac triggering or “unusable”. It was assessed if the durations between triggers appeared reasonable and if they had a strong similarity to other methods. For all videos, the PO was labeled in 12.5% as unusable, the ECG in 58.3%, and the proposed video-based method in 33.3%. To account for conditions during MRA, the videos during stage (3) with MR imaging were analyzed individually and the PO was labeled in 37.5% as unusable, the ECG in 87.5%, and the vPPG method in 37.5%.

**Fig. 11:**
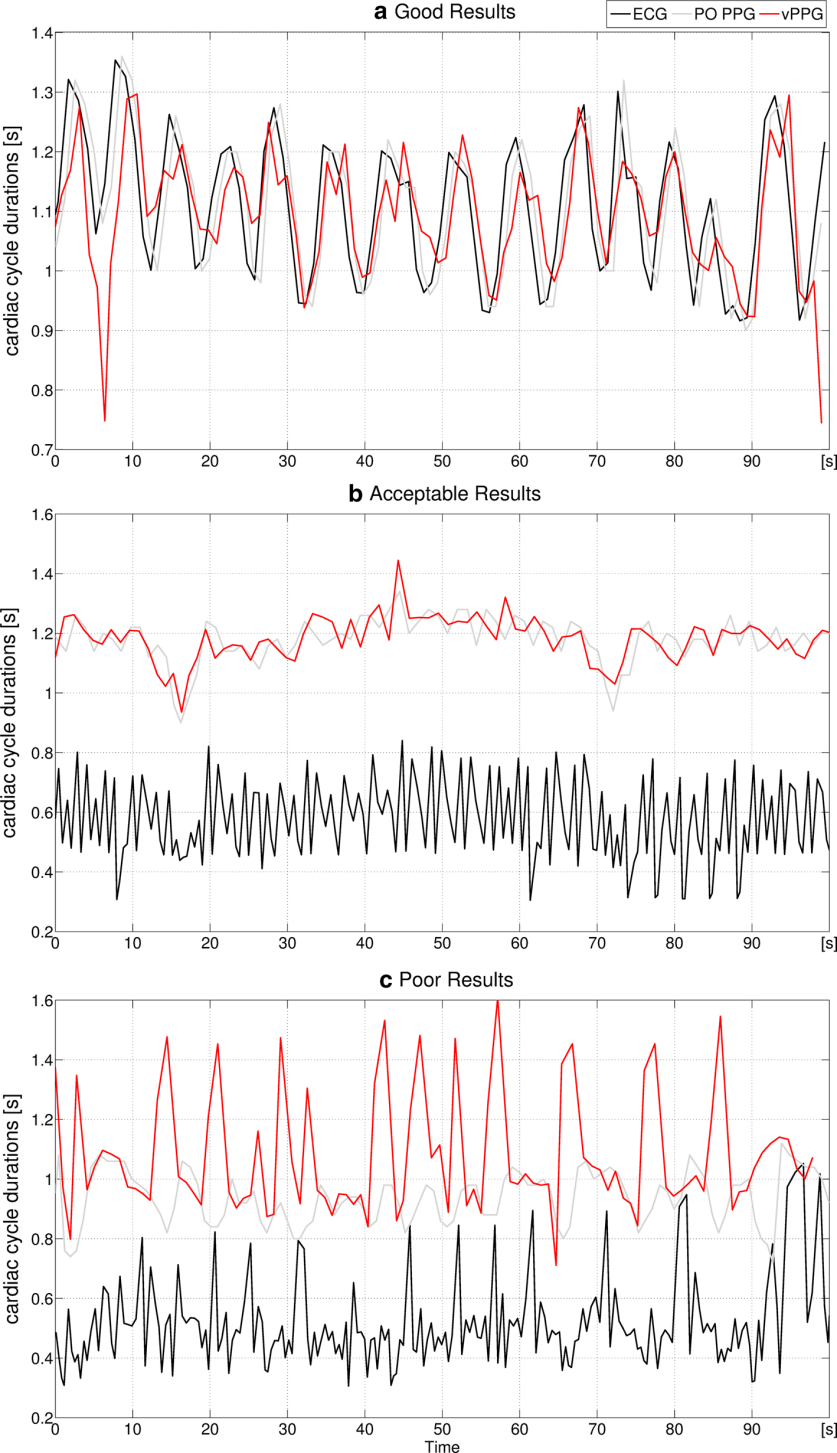
Algorithms performance in detail. Durations of the cardiac cycles between consecutive triggers from ECG, PO, and the vPPG algorithm. Shown here are the results obtained with the videos marked **a**–**c** in Fig. 10

The results of three videos with good (a), acceptable (b), and poor performance (c) of the algorithm, where the latter was classified as unusable, are shown Fig. 11. In (a), results obtained outside the MRI scanner with accurate ECG/PO and vPPG triggering are shown. The vPPG algorithm performs comparably with only minor deviations. (b) Shows in-bore results with many false ECG triggers. The vPPG triggers are not as accurate as in (a), but they outperform ECG and are able to capture trends of the PO triggering, e.g. the downward slope around 18s. (c) Shows poor performance of the algorithm with many missed triggers.

#### Comparison of vPPG- and PO-triggered MRA images and trigger times

**Fig. 12:**
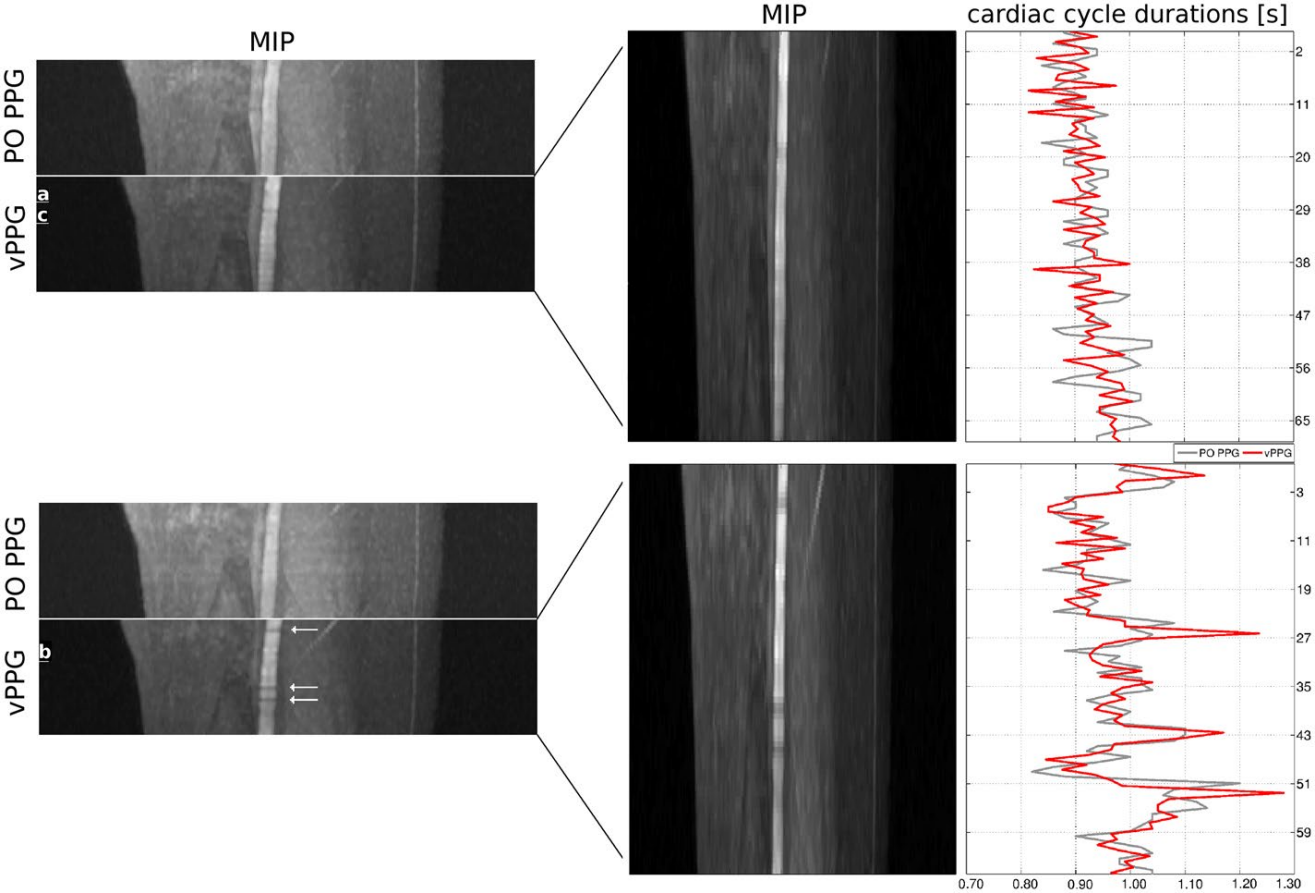
Coronal reformated MRA MIPs zoomed to the right leg of a healthy volunteer. Four MIPs consisting of 30 individual MRA images each were obtained using PO PPG and vPPG triggering. On the* right side* the corresponding trigger-to-trigger times between vPPG triggers (*red curve*) can be seen. As reference, the PO trigger-to-trigger times (*gray curve*) are shown as well. As can be seen, during acquisition of the second MIP, the HRV of the subject was increased. The* arrows* indicate short declines of the vessel signal as a result of inaccurate triggering

The proposed method with optimized algorithm parameters and PO were used to trigger the scanner during MRA of a healthy volunteer. Figure 12 shows MIP images obtained from identical positions using both methods. Both triggering methods were used alternately in order to take HRV of the subject into account. Depending on the current HR of the subject, each MIP acquisition took approximately 70 s. As can be seen, both triggering methods led to a homogeneous and hyperintense delineation of the arteries. Images obtained using vPPG triggering have in general a superior artery-to-background contrast; during the second vPPG-based acquisition, three declines of the vessel signal can be seen.

Figure 12 additionally shows the corresponding trigger-to-trigger times as well as the values from PO PPG triggers that were recorded in parallel but not used for MRI triggering. Visual comparison shows that no triggers were missed by the video-based method during either scan, which allows computation of quantitative measures. Table 2 summarizes the results: during both scans, the subject had normal cardiac activity; however, during the second MRA the sd of cardiac cycle durations, i.e. the subjects HRV, was increased compared to the first scan. Comparing the mean and sd of cardiac cycle durations, i.e. the durations between triggers, shows that the vPPG triggers were more similar to PO triggers during the second experiment. Similarly, the trigger delay and jitter were reduced with a mean trigger delay of 312 ms compared to 401 ms and a trigger jitter of 129 ms compared to 152 ms. Although the vPPG triggers were quantitatively more similar to the PO PPG triggers than in the first scan, three short vessel declines can be seen in the MIP of the second scan.

**Table 2:** Comparison of PO and vPPG triggers during MRA of a healthy volunteer

Scan	Cardiac cycles	Missed cardiac cycles		Cardiac cycle durations (ms)		Trigger delay and jitter (ms)
		PO PPG	vPPG	PO PPG	vPPG	vPPG
1.	73	0	0	931 ± 48	923 ± 42	401 ± 157
2.	74	0	0	975 ± 76	970 ± 79	312 ± 129

Scan (1) is associated with the upper MIP and scan (2) with the lower MIP in Fig. 12. The trigger delay was measured as the mean difference in absolute trigger times between both methods and the trigger jitter as the sd

**Fig. 13:**
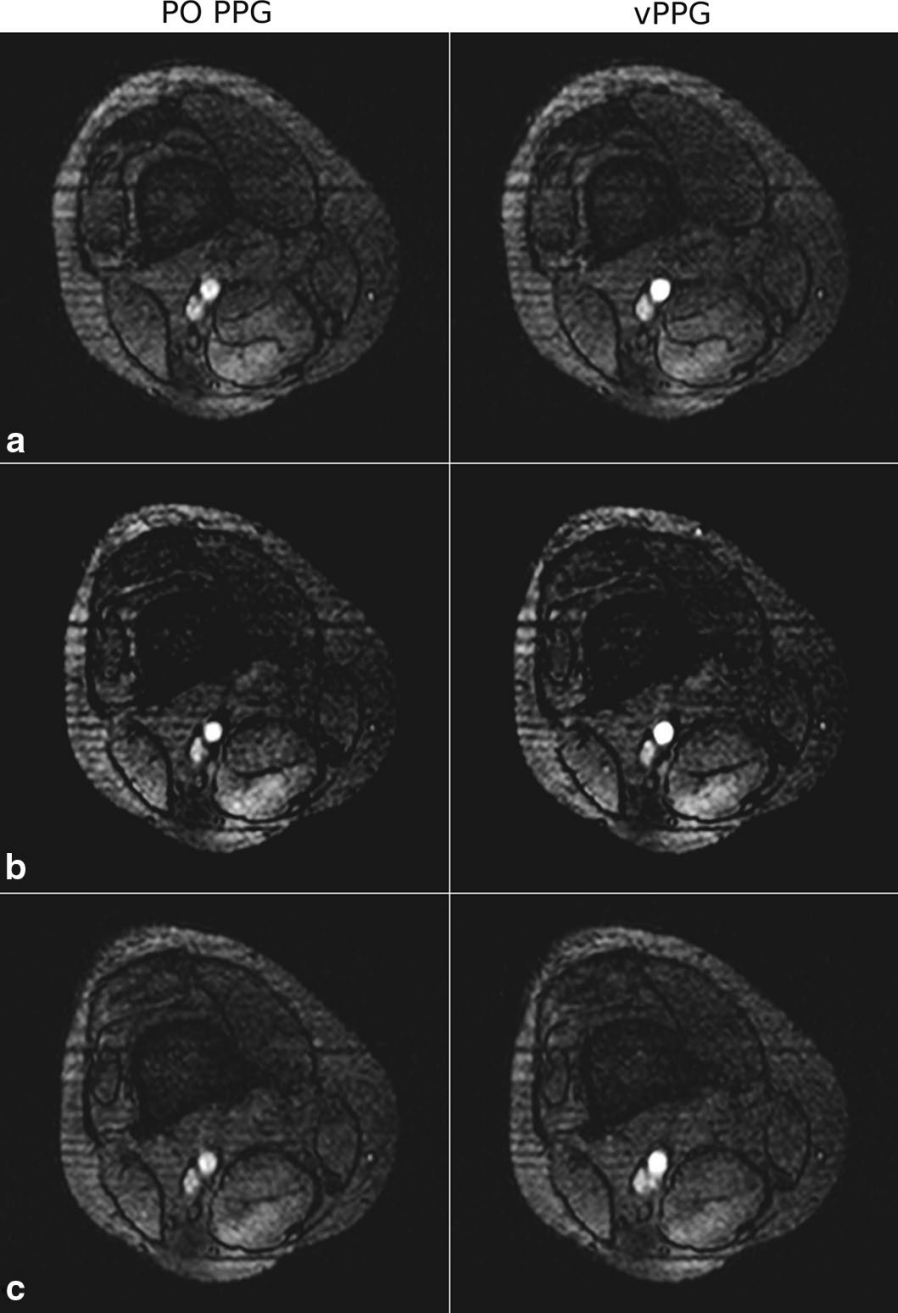
Acquired MRA images of an axial slice obtained during PO and vPPG triggering. In **a** the image acquired using vPPG triggering has a sharply defined vessel compared to the blurred vessel obtained by PO triggering. In **b** the vessel has a sharp outline and high contrast in both images. In **c** the image acquired using vPPG exhibits a blurred vessel signal compared to the sharply defined outline that was obtained by PO triggering

Figure 13 shows the individual MRA images that were marked in Fig. 12. Additionally, images from the same position obtained using PO triggering are shown. As can be seen, the vPPG-triggered images have better (a), equal (b), or inferior (c) image quality compared to the PO PPG-triggered images.

## Discussion and conclusions

In this work, a new cardiac triggering method based on real-time processing of videos captured with an in-bore camera has been introduced and used in the context of ultra-high-field MRA of the lower extremities. To the best of our knowledge, this is the first time video-triggered MR images have been obtained.

**Fig. 14:**
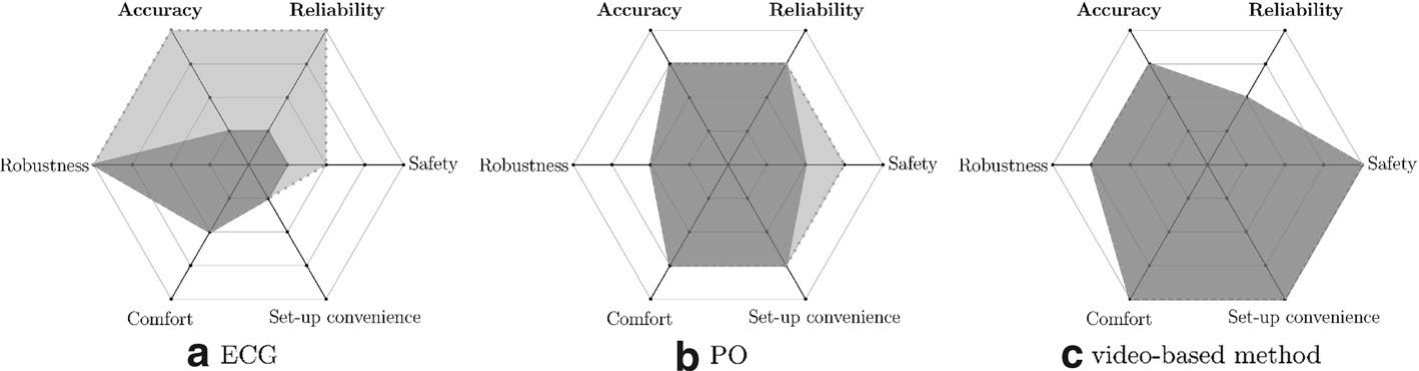
Assessment of triggering methods (**a** ECG, **b** PO, **c** proposed method) for clinical (≤3 T) and high-field MRI (≥7 T). The* gray areas* represent the assessment for clinical MRI and the* black areas* for high-field MRI. Each value is expressed relative to the performance of both other triggering methods in its dimension and ranges linearly from poor performance (*center*) to best performance (*outside border*). The different dimensions are: accuracy (describing time difference between depolarization of the ventricles and issuance of a trigger), reliability (describing reliability independent of environment variables such as field strength, coil activity, illumination etc.), robustness (describing robustness against noise introduced by the patient such as signal loss due to decreasing perfusion, head or finger motion, or increased HRV), comfort (describing patient comfort when the method is applied), safety (describing potential safety hazards from the hardware), and set-up convenience (describing the time and effort it takes to apply the method). *In this work, we have provided data that supports the statements of the figure with respect to accuracy and reliability. The statements with respect to the other dimensions, represent a subjective assessment and are based on current literature and observations made during this work*

In the proposed method, a camera measures skin color variations that contain a subtle PPG signal with a similar waveform as the one measured by contact-based PO but with a significantly smaller SNR. As both methods are based on PPG measurement, the proposed method cannot outperform PO under ideal conditions, but it can overcome shortcomings that occur in its practical application, i.e. restriction of the application area to the finger and progressive loss in signal amplitude with decreasing perfusion of the upper extremities, often observed during long scan times. Additionally, we observed susceptibility to gradient vibrations of the MRI vendor-provided PO Bluetooth module during our experiments. Currently, at clinical field strengths (1.5/3T), ECG is often the preferred triggering method, but its sensitivity to noise due to magnetohydrodynamic interferences is still a major obstacle in the context of ultra-high-field MRI [8]. These drawbacks are reflected in the assessment of both triggering methods summarized in Fig. 14a, b. Due to their reliability (ECG) and robustness (PO) limitations in ultra-high-field MRA, their lack in patient safety and comfort, as well as set-up inconvenience, we began developing the video-based method with the aim to close this gap.

We obtained the following results from experiments:

1. Video noise estimation allows drawing the conclusions that, as described in other publications, illumination is a key factor for obtaining accurate vPPG signals. A video projector was used to provide additional illumination inside the bore, but the SNR in the bore is still inferior to room illumination. As there was no distinct SNR difference between experiments with and without MR scanner activity, we assume that the camera does not seem to be affected by potential artifact sources such as gradient vibrations or excitation pulses.
2. Parameter optimization using synthetic signals showed that there are different optimal *N* values for the synthetic and the non-synthetic signals. This is assumed to be a result from several reasons: It is not certain that a cosine wave is an adequate representation of the skin color intensity changes during one cardiac cycle, the noise characteristics have only been estimated, and aspects such as patient movement or respiration have not been included in the used model. However, the results obtained with synthetic signals underline our assumptions concerning the influence of parameter *N* on the algorithm’s accuracy and it was chosen using the optimization results as a guideline. Optimization of parameter *T* showed that a value of 200ms achieves best results. This underlines that the algorithm is real-time feasible and that reducing the durations until issuance of a trigger due to computation time is not required. However, considering the low number of subjects in our study, we assume that the optimized *T* value provides the best results for the videos at hand but it is not associated to a general property of vPPG signals.
3. Comparing the ECG, PO, and vPPG triggers obtained with optimized parameters from the recorded videos of eight subjects, we were able to show that under good conditions, the vPPG triggers are close to the PO triggers, indicating the potential of the proposed contact-free MRI triggering method. Additionally, the proposed method is able to outperform ECG, which is distorted by high-field artifacts inside the MRI-bore. However, in 3/8 videos, our method was not able to perform satisfactory because of a too low SNR of the vPPG signal.
4. Comparison of MRA images obtained by vPPG and PO triggering in one subject showed that our method is able to produce similar image quality. However, we observed that during increased HRV, an artifact associated with inaccurate triggering [12] appeared in the MRA images. This is interesting because the triggers were quantitatively more similar to PO than during another scan that contained no artifacts. This is most probably a result of a higher susceptibility to artifacts of the MRA procedure during increased HRV and the fact that our algorithm estimates the time until a trigger is sent based on past values. This prediction becomes inaccurate when the duration of the current cardiac cycle is not similar to the durations of the preceding cardiac cycles. Additionally, in both MRA scans there was a relatively large trigger delay <300 ms, which apparently did not negatively affect image quality and is mostly a result of the chosen *T* value. An assessment of the trigger jitter value is difficult as we could not find any values from literature concerning the influence of trigger jitter on MRA image quality.

Based on these results, in future work (1) the limitations during video acquisition need to be addressed to increase reliability: analogously to the works by Maclaren et al. [27], we used a monochrome camera usually used for patient monitoring with a not optimal spectral sensitivity with regard to the wavelength of the PPG signal. In future work, another configuration could be used, for example the green channel of a RGB camera as hinted at by Verkruysse et al. [17]. Additionally, the low illumination inside the MRI bore reduces video quality significantly. Keeping subject comfort in mind, we did not apply LED illumination but instead a video projector which is apparently not sufficient. Maclaren et al. set up a white LED next to the camera [27], which may provide better illumination. Kamshilin et al. used one [11] operating at the designated wavelength of 525 nm which could additionally increase SNR.

(2) Additionally, in future work the influence of HRV on the method’s robustness, and thus on MRA image quality, has to be analyzed in more detail by performing MRA in a larger group of subjects. If an increased HRV turns out to always bias the results of the proposed method, a camera with a higher FPS or a modified algorithm that adapts to the HRV of the subject could be applied to reduce this effect. Additionally, if the SNR of the vPPG signal could be increased significantly by the previously mentioned approaches, the raw vPPG could be processed in the time domain. This would allow triggering based on signal slope without averaging over previously acquired frames as in the current algorithm which should minimize the influence of HRV to a level similar to PO.

Furthermore, in future work (3) synthetic signals should be generated using a more physiologically based model and (4) it has to be examined if the relatively large trigger delay has a physiological reason, i.e. if the vPPG PTT is different to the PO PPG PTT because of different durations until the blood arrives in the forehead or finger. However, initial experiments in the literature [20] indicate that this is not the case and therefore the accuracy of PO and vPPG should be similar.

Other aspects that can be considered in future work are: (1) the influence of skin color on the performance of our method that could not be assessed in this work although our study population included subjects from different ethnic origins. Therefore, we leave a thorough evaluation with a larger and more diverse study population for future work. (2) The impact of subject motion on the algorithms performance. As expected, low-grade motion of healthy and cooperative subjects did not influence the algorithm’s performance significantly because of signal averaging in the ROI. For cooperative patients, we expect similar results, especially since in clinical practice a patient’s head is commonly stabilized using a head rest. In other cases, the accuracy of the proposed method would most likely decrease significantly.

Based on the obtained data in this work, Fig. 14c shows an assessment of the proposed method compared to ECG and PO. The results indicate that we were able to close part of the mentioned gap in ultra-high-field triggering with the developed method, that the method is technically feasible, and it holds a potential for accuracy comparable to PO. However, for more accurate results and eventual clinical application, the reduced reliability due to low SNR has to be increased by addressing the above mentioned limitations during video acquisition.
